# The Development of a Skin Cancer Classification System for Pigmented Skin Lesions Using Deep Learning

**DOI:** 10.3390/biom10081123

**Published:** 2020-07-29

**Authors:** Shunichi Jinnai, Naoya Yamazaki, Yuichiro Hirano, Yohei Sugawara, Yuichiro Ohe, Ryuji Hamamoto

**Affiliations:** 1 Department of Dermatologic Oncology, National Cancer Center Hospital, 5-1-1 Tsukiji, Chuo-ku, Tokyo 104-0045, Japan; nyamazak@ncc.go.jp; 2 Preferred Networks, 1-6-1 Otemachi, Chiyoda-ku, Tokyo 100-0004, Japan; hirano@preferred.jp (Y.H.); suga@preferred.jp (Y.S.); 3 Department of Thoracic Oncology, National Cancer Center Hospital, 5-1-1 Tsukiji, Chuo-ku, Tokyo 104-0045, Japan; yohe@ncc.go.jp; 4 Division of Molecular Modification and Cancer Biology, National Cancer Center Research Institute, 5-1-1 Tsukiji, Chuo-ku, Tokyo 104-0045, Japan; 5 Cancer Translational Research Team, RIKEN Center for Advanced Intelligence Project, 1-4-1 Nihonbashi, Chuo-ku, Tokyo 103-0027, Japan

**Keywords:** melanoma, skin cancer, artificial intelligence (AI), deep learning, neural network

## Abstract

Recent studies have demonstrated the usefulness of convolutional neural networks (CNNs) to classify images of melanoma, with accuracies comparable to those achieved by dermatologists. However, the performance of a CNN trained with only clinical images of a pigmented skin lesion in a clinical image classification task, in competition with dermatologists, has not been reported to date. In this study, we extracted 5846 clinical images of pigmented skin lesions from 3551 patients. Pigmented skin lesions included malignant tumors (malignant melanoma and basal cell carcinoma) and benign tumors (nevus, seborrhoeic keratosis, senile lentigo, and hematoma/hemangioma). We created the test dataset by randomly selecting 666 patients out of them and picking one image per patient, and created the training dataset by giving bounding-box annotations to the rest of the images (4732 images, 2885 patients). Subsequently, we trained a faster, region-based CNN (FRCNN) with the training dataset and checked the performance of the model on the test dataset. In addition, ten board-certified dermatologists (BCDs) and ten dermatologic trainees (TRNs) took the same tests, and we compared their diagnostic accuracy with FRCNN. For six-class classification, the accuracy of FRCNN was 86.2%, and that of the BCDs and TRNs was 79.5% (*p* = 0.0081) and 75.1% (*p* < 0.00001), respectively. For two-class classification (benign or malignant), the accuracy, sensitivity, and specificity were 91.5%, 83.3%, and 94.5% by FRCNN; 86.6%, 86.3%, and 86.6% by BCD; and 85.3%, 83.5%, and 85.9% by TRN, respectively. False positive rates and positive predictive values were 5.5% and 84.7% by FRCNN, 13.4% and 70.5% by BCD, and 14.1% and 68.5% by TRN, respectively. We compared the classification performance of FRCNN with 20 dermatologists. As a result, the classification accuracy of FRCNN was better than that of the dermatologists. In the future, we plan to implement this system in society and have it used by the general public, in order to improve the prognosis of skin cancer.

## 1. Introduction

Skin cancer is the most common malignancy in Western countries, and melanoma specifically accounts for the majority of skin cancer-related deaths worldwide [1]. In recent years, many skin cancer classification systems using deep learning have been developed for classifying images of skin tumors, including malignant melanoma (MM) and other skin cancer [2]. There are reports that their accuracy was at the same level as or higher than that of dermatologists [3,4,5].

The targeted detection range of previous reports was from only malignant melanoma to the entire skin cancer. Image data used for machine learning were clinical images and dermoscopic images. Up to now, there has been no report of training a neural network using clinical image data of pigmented skin lesions and evaluating the accuracy of the system to classify skin cancer, such as MM and basal cell carcinoma (BCC). When developing a system, it is important to determine the appropriate endpoints according to the type of skin tumor to be targeted, as well as the method of imaging. When new patients come to a medical institution with skin lesions as the chief complaint, they are generally concerned not about whether they are malignant melanomas, but whether they are skin cancers. Therefore, there is a need to develop a system that can also detect other skin tumors that have a pigmented appearance similar to malignant melanoma. There are also erythematosus skin malignancies, such as mycosis fungoides [6], extramammary Paget’s disease [7], and actinic keratosis [8], which is a premalignant tumor of squamous cell carcinoma. It is often difficult to distinguish these cancers from eczema. Since we are focusing on the detection of brown to black pigmented skin lesions, including MM, we have excluded these cancers in this study.

In recent years, with the progress of machine learning technology mainly on deep learning, the expectations of artificial intelligence has been increasing, and research on its medical application has been actively progressing [9,10,11,12]. In the present study, we used the faster, region-based convolutional neural network (Faster R-CNN, or FRCNN) algorithm, which is a result of merging region proposal network (RPN) and Fast R-CNN algorithms, into a single network [13,14]. The pioneering work of region-based target detection began with the region-based convolutional neural network (R-CNN), including three modules: regional proposal, vector transformation, and classification [15,16]. Spatial pyramid pooling (SPP)-net optimized the R-CNN and improved detection performance [16,17]. Fast R-CNN combines the essence of SPP-net and R-CNN, and introduces a multi-task loss function, which is what makes the training and testing of the whole network so functional [16,18]. FRCNN merges RPN and Fast R-CNN into a unified network by sharing the convolutional features with “attention” mechanisms, which greatly improves both the time and accuracy of target detection [13,16]. Indeed, FRCNN has shown higher detection performance in the biomedical filed than other state-of-the-art methods, such as support vector machines (SVMs), visual geometry Group-16 (VGG-16), single shot multibox detectors (SSDs), and you only look once (YOLO), in terms of time and accuracy [19,20,21]. In particular, FRCNN has achieved the best performance for diabetic foot ulcer (DFU) detection; the purpose of the DFU study was similar to our research goal [21]. Therefore, we ultimately chose the FRCNN architecture in this study. Moreover, in the medical science field, transductive learning models have widely been used in addition to supervised learning models [22,23]. Meanwhile, given that diagnosis is a medical practice and requires authorized training data by medical doctors, we chose supervised learning in the present study. 

Importantly, many mobile phone applications that can detect skin cancers have been developed and put on the market [24,25,26]. In those applications, skin cancer detection is performed using smartphone camera images rather than the magnified images of dermoscopy, which is commonly used by dermatologists in medical institutions. Our goal is to develop a skin cancer detection system that can be easily used by people who are concerned about the possibility that the skin lesion is cancers. Therefore, in this study, we developed a neural network-based classification system using clinical images rather than dermoscopic images. We evaluated the accuracy of the system and asked dermatologists to take the same test, in order to compare the accuracy with the deep learning system we developed.

## 2. Materials and Methods

### 2.1. Patients and Skin Images

This study was approved by the Ethics Committee of the National Cancer Center, Tokyo, Japan (approval ID: 2016-496). All methods were performed in accordance with the Ethical Guidelines for Medical and Health Research Involving Human Subjects; with regard to the handling of data, we followed the Data Handling Guidelines for the Medical AI project. Of approximately 120,000 clinical images taken from 2001 to 2017 at the Department Dermatologic Oncology in the National Cancer Center Hospital, we extracted 5846 clinical images of brown to black pigmented skin lesions from 3551 patients. The clinical images were taken by digital cameras and stored as digital images. Additionally, we confirmed that all images were of sufficient quality that dermatologists could diagnose (Supplementary Table S1). The target diseases are malignant tumors (MM and BCC) and benign tumors (nevus, seborrheic keratosis (SK), senile lentigo (SL) and hematoma/hemangioma (H/H)). The breakdown of the extracted images was 1611 MM images (from 710 patients), 401 BCC images (from 270 patients), 2837 nevus images (from 1839 patients), 746 SK images (from 555 patients), 79 SL images (from 65 patients), and 172 H/H images (from 147 patients). All malignant tumors were biopsied and diagnosed histopathologically. Benign tumors were diagnosed clinically using dermoscopy, and those cases that were still difficult to differentiate were biopsied to make confirmed diagnosis. All of the images were taken with digital, single-lens reflex cameras, which had at least 4.95 million pixels, a macro lens, and macro ring flash. No dermoscopic images were included in this study. Out of the 3551 patients, we randomly selected 666 patients, and picked one image per patient for the test dataset. The remaining 4732 images from 2885 patients were used for training. The breakdown of the 666 images of the test dataset was 136 MM images (from 136 patients), 44 BCC images (from 44 patients), 349 nevus images (from 349 patients), 96 SK images (from 96 patients), 15 SL images (from 15 patients), and 26 H/H images (from 26 patients). The breakdown of the 4732 images of the training dataset was 1279 MM images (from 566 patients), 344 BCC images (from 222 patients), 2302 nevus images (from 1474 patients), 606 SK images (from 451 patients), 62 SL images (from 51 patients), and 139 H/H images (from 121 patients). We gave bounding-box annotations (where and what class each lesion is) to all the images, and a dermatologist (S.J.) confirmed their validity.

**Figure 1:**
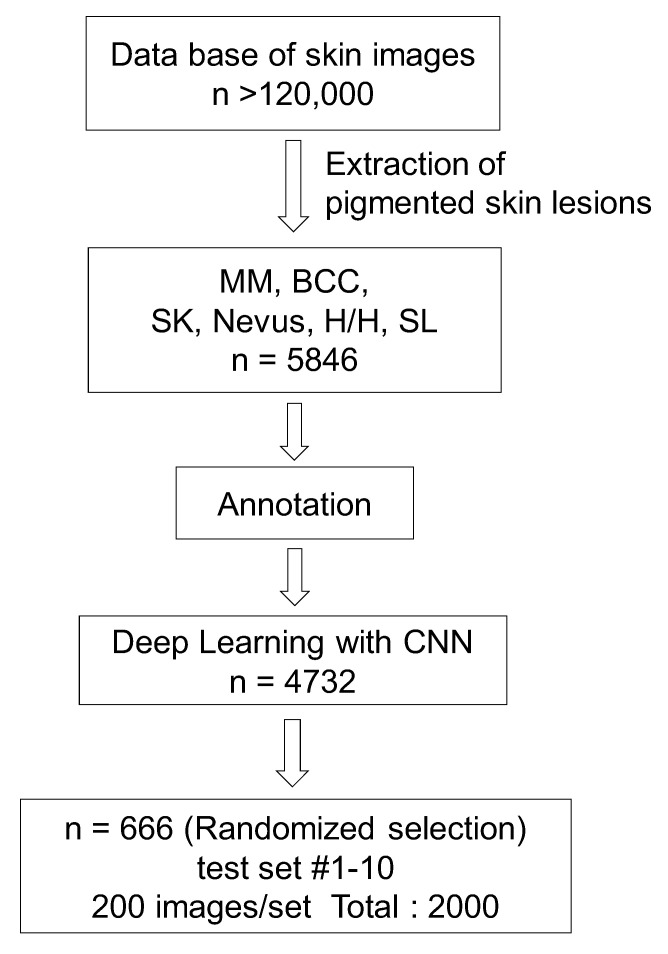
Flow diagram of this study: extracting the pictures of pigment lesions, annotation of lesions in images, deep learning with a convolutional neural network (CNN), and evaluation by the test dataset.

To reduce each dermatologist’s burden, we randomly sampled 200 images from 666 images and created tests of 10 patterns, so that each image was selected at least three times (200 images × 10 sets = 2000 images; 2000 ÷ 666 patients = 3). Thus, each test consisted of 200 images. The whole flow diagram is shown in Figure 1.

### 2.2. Training of a Deep Learning Model

With regard to the deep learning architecture, we placed the highest priority on accuracy and rapidity in choosing a model, because accurate and prompt classification is required in the medical field. As a result of various comparison, we finally selected the FRCNN; this model stably showed high classification accuracy, robustness, and rapidity [13,14,27,28,29]. Then, we trained an FRCNN model with the training dataset. We used Visual Geometry Group-16 (VGG-16) [30] as its backbone, and a Momentum stochastic gradient descent (SGD) [31] optimizer with learning rate of 1 × 10^−3^ and momentum of 0.9. We used weight decay of 5 × 10^−4^ and the batch size was 4. The model was trained for 100 epochs, and the learning rate was decreased by a factor of 10 after 40 and 80 epochs finished. Images of BCC, SL, and H/H were twice oversampled during training. Horizontal flip, random distort [32], 90 and 180 degree rotations, random cropping, and zoom were used for data augmentation. We used Chainer [33], ChainerCV [34], and Cupy [35] for the implementation of our network.

### 2.3. Test-Time Augmentation

During inference, we used test-time augmentation. Specifically, an input image underwent transformations of horizontal flip (two patterns); 72 degree rotations (five patterns); and 1×, 1.2×, or 1.4× zoom (three patterns), yielding 30 patterns of images in total. Predictions were made on all 30 images, and the predicted region with the highest confidence among all predictions was selected as the final prediction for that input image.

### 2.4. Model Validation and Verification

Our model (FRCNN), 10 board-certified dermatologists (BCDs), and 10 trainees (TRNs) were assessed using 10 patterns of tests, and we compared their performances. We compared the results in two patterns: a six-class classification (judge what class each sample is) and a two-class classification (judge whether each sample is benign or malignant). We calculated the accuracy for both six- and two-class classifications by the following formula: accuracy (%) = (total number of correct predictions)/(total number of all samples) × 100. For two-class classification, we also calculated sensitivity, specificity, false negative rates, false positive rates, and positive predictive values. The accuracy of two- and six-class classification was compared with the equivalent of each other using a paired *t*-test, and *p*-values < 0.05 were considered significant.

## 3. Results

### 3.1. Six-Class Classification of FRCNN, BCDs, and TRNs

**Figure 2:**
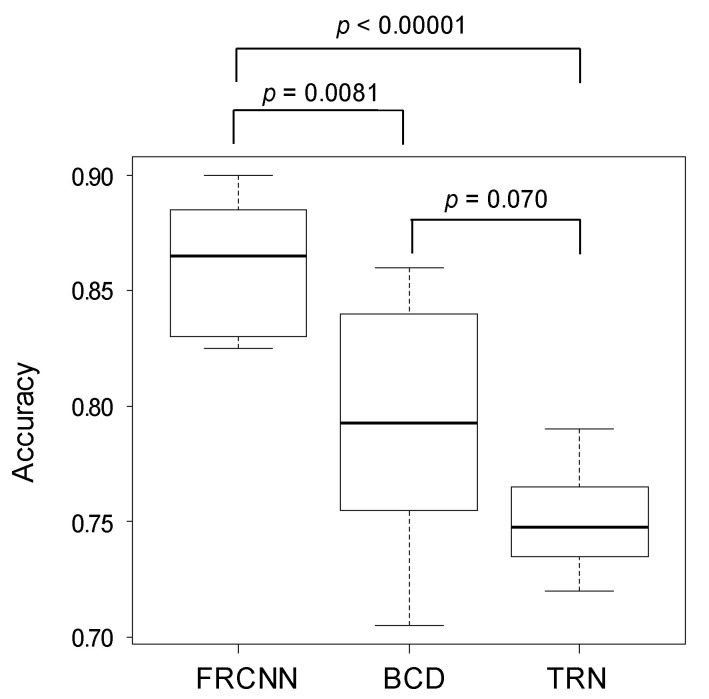
The accuracy of six-class classification by FRCNN, BCDs, and TRNs. In six-class classification, the accuracy of the FRCNN surpassed that of BCDs and TRNs.

The results (200 questions × 10 tests) of six-class classification of FRCNN, BCD, and TRN are shown in Table 1. The accuracy of the six-class classification of FRCNN was 86.2% (1724/2000), while those of BCD and TRN were 79.5% (1590/2000) and 75.1% (1502/2000), respectively. The accuracy of six-class classification of each examinee is shown in Table 2. Except for test #2, FRCNN had higher accuracy than the dermatologists. The standard deviation of the accuracy of six-class classification of FRCNN was 2.80%, and that of the dermatologists was 4.41%. The accuracy of six-class classification by FRCNN (86.2 ± 2.95%) was statistically higher than that of BCD (79.5 ± 5.27%, *p* = 0.0081) and TRN (75.1 ± 2.18%, *p* < 0.00001). The accuracy of six-class classification by BCD was not statistically higher than that of TRN (*p* = 0.070) (Figure 2).

### 3.2. Two-Class Classification of FRCNN, BCDs, and TRNs

**Figure 3:**
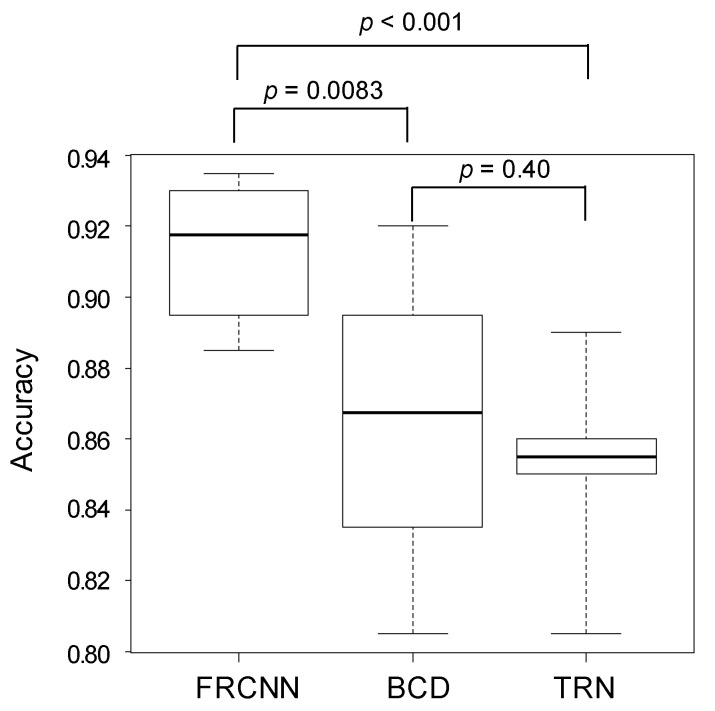
The accuracy of two-class classification (benign or malignant) by FRCNN, BCDs, and TRNs. The accuracy of the FRCNN surpassed that of the BCDs and TRNs.

The results of two-class classification (benign or malignant) of FRCNN, BCDs, and TRNs are shown in Table 3. Malignant tumors include MM and BCC, and benign tumor includes nevus, SK, SL, and H/H. The accuracy of two-class classification of the FRCNN was 91.5% (1829/2000), while those of BCDs and TRNs were 86.6% (1829/2000) and 85.3% (1705/2000), respectively. The accuracy of two-class classification by the FRCNN (91.5 ± 1.79%) was also statistically higher than that of BCDs (86.6 ± 4.01%, *p* = 0.0083) and TRNs (85.3 ± 2.18%, *p* < 0.001). The accuracy of two-class classification by BCD was not statistically higher than that of the TRNs (*p* = 0.40) (Figure 3).

### 3.3. Two-Class Classification of FRCNN, BCDs, and TRNs

The accuracy of six-class classification of each examiner is shown in Table 4. BCDs had the highest accuracy in test #2, and the BCDs and FRCNN had the same accuracy in test #6. In all the tests other than #2 and #6, FRCNN had the highest accuracy among all examiners. The standard deviation of the accuracy of two-class classifications of FRCNN was 1.69%, and those of BCDs and TRNs were 9.79% and 3.13%, respectively. 

### 3.4. Summary of Classification Conducted by FRCNN, BCDs, and TRNs

We show the summary of the classification accuracy, sensitivity, specificity, false negative rates, false positive rates, and positive predictive values by FRCNN, BCDs, and TRNs in Table 5. FRCNN achieved highest accuracy and sensitivity. On the other hand, BCDs achieved the highest specificity. The false negative rates of all of them are almost the same, but the false positive rates of the dermatologists (BCDs: 13.4%; TRNs: 14.1%) were higher than that of the FRCNN (5.5%). The false positive rates of the dermatologists were higher than that of the FRCNN, and the positive predictive values of them were lower (BCDs: 70.5%, TRNs: 68.5%) than that of the FRCNN (84.7%).

## 4. Discussion

In this study, we developed a classification system by deep learning for brown to black pigmented skin lesions, as the target disease. Then, the same test dataset was used for examining 20 dermatologists, and the accuracy of them was compared with that of the FRCNN. The results showed that only one out of 20 dermatologists had higher accuracy than the FRCNN in six-class classification. The skin tumor classification system using deep learning showed better results in both six- and two-class classification accuracy than BCDs and TRN dermatologists. Many similar tests have been reported in previous research [3,36,37], and it is considered that the machine learning algorithm has reached dermatologist-level accuracy in skin lesion classification [4,5,36]. In the present study, although the FRCNN and the dermatologists had similar results in terms of sensitivity, false positive rates were BCDs: 13.4%, TRNs: 14.1%, and FRCNN: 5.5%. It is likely that when the dermatologists were uncertain whether skin lesions were malignant or benign, they might tend to diagnose them as malignant. The dermatologists had higher false positive rates, and the positive predictive values were 70.5% by the BCDs and 68.5% by the TRNs, and lower than 84.7% by the FRCNN. False negative rates have been regarded as more important than false positive rates in such diagnostic systems for malignancy, but false positive rates must be carefully monitored. This is because false positive predictions give users unwanted anxiety. In addition, although the results of the dermatologists varied, the results of the FRCNN showed less variation. Brinker et al. reported that CNNs indicated a higher robustness of computer vision compared to human assessment for clinical image classification tasks [3]. This is due to the lack of concentration during work, which is unique to humans. It is considered that there may be differences in clinical ability depending on the years of experience of dermatologists.

We think that it is important to determine how to implement these results socially after system development and connect them to users’ benefit. Depending on the concept of system development, the endpoint and the type of image data required for the development will change. For example, if the person who uses the system is a doctor, highly accurate system development closer to a confirmed diagnosis will be required. Training neural networks that can detect cancers from dermoscopic images will be also in need. However, for in-hospital use there is already a diagnostic method: biopsy. Biopsy is a method of taking a part of skin tissue and making a pathological diagnosis. Through a biopsy, it is possible to make an almost 100% diagnosis (confirmed diagnosis). Moreover, the procedure of biopsy takes only about 10 min. It is an advantage of dermatologists to be able to perform biopsy more easily than other department doctors, and it seems that there is no room for new diagnostic functions of any diagnostic imaging systems in medical institutions. On the other hand, when considering their use by the general public outside medical institutions, it is difficult to fully demonstrate their diagnostic performance. This is because the reproducibility of shooting conditions cannot be ensured, and the shooting equipment is different. Therefore, when using an imaging system outside medical institutions, it may be better to use the system to call attention to skin cancer rather than focus on improving diagnostic performance. Also, no one can say that the accuracy of the system needs to be improved when it is used outside the medical institution.

Mobile phone applications that can detect skin cancer and malignant melanoma have already been launched in countries around the world [24]. However, usage of such applications for the self-assessment of skin cancer has been problematic, due to the lack of evidence on the applications’ diagnostic accuracy [38,39]. In addition to the problem of low accuracy, there is also a problem that they sometimes cannot recognize images well [25]. The reason is that the quality of images may be lower, and that there is more variability in terms of angles, distances, and the characteristics of the smartphone [40]. If the shooting conditions are bad, the accuracy is naturally low. This is an unavoidable task in terms of social implementation, in which the users are general public and the device used is a mobile phone camera. The main risk associated with the usage of mobile phone application software by general public is that malignant tumor may be incorrectly classified as low-risk, and its diagnosis and appropriate treatment are delayed. To solve these problems. and to improve the accuracy of the application over time, a large dataset is necessary to cover as many image-taking scenarios, as well as other information (i.e., ages, position of the primary lesion, the period time from first awareness to visit a dermatologist, etc.) as possible. However, it takes a lot of effort to create such a dataset. Udrea et al. have succeeded in improving accuracy by changing the learning method and training, with a large number of skin tumor images taken with a mobile phone [40]. We must be careful to make users fully aware that mobile phone application software is a system that also has the negative aspects. In fact, SkinVision, an application for detecting skin cancers, also states that “assessment does not intend to provide an official medical diagnosis, nor replace visits to a doctor [40].”

We are also planning a future social implementation system of skin cancer classification to be used by the general public, with wearable devices, such as mobile phones. The original concept is to have early skin cancer detection, early treatment, and improved prognosis of skin cancer patients. In Japan, the incidence of skin cancer is lower than in Western countries, and its awareness is also low. The proportion of advanced stage cases of melanoma is higher than in Europe and the United States [41,42]. As a result, many patients tend to have poor outcomes. In recent years, the prognosis of melanoma has been improved by new drugs, such as immune checkpoint inhibitors and molecular-targeted therapy [43], but at the same time, the problem of rising medical costs has arisen [44]. In Japan, there is no official skin cancer screening, and there is no intervention that can be performed early for the entire Japanese population. Additionally, since melanoma is one of the rarer skin cancers for Japanese people, it is not well-recognized, and people tend not to see a dermatologist at the early stages [43]. The average period from first awareness to visit of Japanese melanoma patients was 69.5 months; the median was 24 months. In other countries, the median period is reported to be 2 months to 9.8 months, which is very different from the reports in Japan [45,46,47,48]. The rate of late-stage is high, due to the longer period from first awareness to visit. Because the stage of disease at the first visit is highly related to the prognosis of skin cancer [49], early detection of skin cancer is very important. If skin cancer is detected at an early stage, it will be easier to treat, and the prognosis will be much better [50]. We think that an intervention that shortens the period from awareness to visit is essential for improving the skin cancer prognosis. Some mobile phone application software that is on the market may have diagnosed skin cancers that were not diagnosed as skin cancer by dermatologists, which helps in the early detection and treatment of skin cancer [38]. In the future, we think that the intervention of skin image diagnostic application software, as described above, can solve various problems, such as improving the prognosis of skin cancer and reducing the treatment costs. Also, by reducing the waiting time for patients and unnecessary visits to outpatient clinics, and facilitating consultations, medical treatment will be efficient [40]. It would be of great value if such an image diagnosis system actually improved the prognosis after social implementation. Such application software has not appeared yet, and we hope we can create such an application in the future.

There are several limitations to this study. First, although all malignant tumors were biopsied and diagnosed histopathologically, benign tumors were confirmed as benign using biopsy, or for those not excised were deemed clinically benign. Second, the neural network was trained using clinical images of brown to black pigmented skin lesions from only our institution, and biases may exist in those data (e.g., portion of disease, type of camera). It will be necessary for future work to check whether the neural network generalizes well with images taken outside our institution. Third, in the present study, we showed only the ability of judging clinical images, but in routine medical care, human medical doctors make a definitive diagnosis by taking biopsies and other clinical information into consideration. Therefore, it is risky to judge that artificial intelligence (AI) is superior to human medical doctors based on this study. Further validation is essential; we need to make a careful judgment on how to implement our findings in society. In addition, this is only the first step, and there is no doubt that large-scale verification will be required as the next step, according to the suitable social implementation method. Lastly, although we used the FRCNN architecture in the present study, we need to carefully choose the best method for achieving our goal, because deep learning technologies have recently been progressing massively [51]. In particular, FRCNN has been reported to have difficulty identifying objects from low-resolution images, due to its weak capacity to identify local texture [52]. We plan to improve the algorithm appropriately, according to the direction of our social implementation.

## 5. Conclusions

We have developed a skin cancer classification system for brown to black pigmented skin lesions using deep learning. The accuracy of the system was better than that of dermatologists. It successfully detected not only malignant melanoma, but also basal cell carcinoma. System development that fits the needs of society is important. We would like to seek the best method for the early detection of skin cancer and improvement of the prognosis.

## Figures and Tables

**Table 1:** The results of six-class classification of the faster, region-based CNN (FRCNN); board-certified dermatologists (BCDs); and trainees (TRNs). Gray cells indicate correct answers.

FRCNN								
Prediction								
True diagnosis		MM	BCC	Nevus	SK	H/H	SL	Total
	MM	327	9	48	21	0	3	408
	BCC	6	108	12	6	0	0	132
	Nevus	42	6	967	30	3	0	1048
	SK	21	9	36	223	0	0	289
	H/H	3	0	18	0	57	0	78
	SL	0	0	0	3	0	42	45
	Total	399	132	1081	283	60	45	2000
**BCDs**								
**Prediction**								
True diagnosis		MM	BCC	Nevus	SK	H/H	SL	Total
	MM	340	12	22	26	3	5	408
	BCC	10	104	3	14	1	0	132
	Nevus	131	11	823	68	11	4	1048
	SK	18	24	17	225	0	5	289
	H/H	9	1	6	1	61	0	78
	SL	0	1	0	7	0	37	45
	Total	508	153	871	341	76	51	2000
**TRNs**								
**Prediction**								
True diagnosis		MM	BCC	Nevus	SK	H/H	SL	Total
	MM	327	15	42	12	8	4	408
	BCC	22	87	6	12	5	0	132
	Nevus	136	17	812	57	20	6	1048
	SK	26	17	37	191	1	17	289
	H/H	8	1	16	2	51	0	78
	SL	1	0	3	7	0	34	45
	Total	520	137	916	281	85	61	2000

MM: malignant melanoma; BCC: basal cell carcinoma; SK: seborrheic keratosis; H/H: hematoma/hemangioma; SL: senile lentigo.

**Table 2:** The accuracy of six-class classification for each examinee. The best accuracy for each test (test #1–10) is shown in gray.

TEST #	FRCNN	BCD	TRN
1	90.00%	84.00%	76.50%
2	82.50%	86.00%	72.00%
3	84.50%	83.50%	74.50%
4	90.00%	79.00%	74.50%
5	83.00%	78.00%	73.00%
6	86.50%	85.50%	75.00%
7	88.00%	70.50%	79.00%
8	86.50%	79.50%	75.00%
9	82.50%	73.50%	78.00%
10	88.50%	75.50%	73.50%

**Table 3:** The results of two-class classification (benign or malignant) of the FRCNN, BCDs, and TRNs. Gray cells indicate correct answers.

FRCNN				
	Prediction			
		malignant	benign	Total
True diagnosis	malignant	450	90	540
	benign	81	1379	1460
	Total	531	1469	2000
**BCDs**				
	**Prediction**			
		malignant	benign	Total
True diagnosis	malignant	466	74	540
	benign	195	1265	1460
	Total	661	1339	2000
**TRNs**				
	**Prediction**			
		malignant	benign	Total
True diagnosis	malignant	451	89	540
	benign	206	1254	1460
	Total	657	1343	2000

**Table 4:** The accuracy of two-class classification for each examinee. The best accuracy for each test (test #1–10) is shown in gray. The accuracy of the BCDs was the best in test #2. In test #6, the BCDs and FRCNN achieved the same accuracy.

TEST #	FRCNN	BCD	TRN
1	93.50%	89.50%	85.00%
2	88.50%	92.00%	86.00%
3	91.00%	89.00%	85.00%
4	93.50%	87.00%	80.50%
5	89.50%	84.50%	85.50%
6	91.50%	91.50%	85.50%
7	92.50%	83.50%	89.00%
8	92.00%	86.50%	86.50%
9	89.50%	81.50%	86.00%
10	93.00%	80.50%	83.50%

**Table 5:** Summary of classification accuracy, sensitivity, specificity, false negative rates, false positive rates, and positive predictive values by the FRCNN, BCDs, and TRNs.

	FRCNN	BCDs	TRNs
Accuracy (six classes)	86.2	79.5	75.1
Accuracy (two classes)	91.5	86.6	85.3
Sensitivity	83.3	86.3	83.5
Specificity	94.5	86.6	85.9
False negative	16.7	13.7	16.5
False positive	5.5	13.4	14.1
Positive predictive value	84.7	70.5	68.5

## Supplementary Materials

The following are available online at https://www.mdpi.com/2218-273X/10/8/1123/s1, Table S1: Information of the digital cameras, which took the skin images in this study.
